# 15th Anniversary of Pharmaceutics—Advances in Process and Formulation Modeling

**DOI:** 10.3390/pharmaceutics16081056

**Published:** 2024-08-11

**Authors:** Ecevit Bilgili, Sadegh Poozesh

**Affiliations:** 1 Otto H. York Department of Chemical and Materials Engineering, New Jersey Institute of Technology, Newark, NJ 07102, USA; 2 Mechanical Engineering Department, Tuskegee University, Tuskegee, AL 36088, USA; sadegh.poozesh@gmail.com

The modeling of processes and formulations significantly enhances product development in the pharmaceutical industry. An important element of the Quality-by-Design (QbD) framework of product development, aligned with the expectations outlined in the International Council on Harmonization (ICH) guidance Q8(R2), Q9, and Q10 [1], is the development of a fundamental mechanistic understanding of manufacturing processes, underpinned by a science- and risk-based approach. Modeling helps scientists, formulators, and engineers gain these fundamental insights [2], leading to a streamlined and faster development of pharmaceutical products. Various types of models are available to scientists and engineers [2,3,4], such as Computational Fluid Dynamics (CFD), Population Balance Model (PBM), Discrete Element Model (DEM), molecular simulations via Density Functional Theory (DFT), and statistics-based models. Readers will find the latest research on the modeling of various pharmaceutical processes and formulations in this Special Issue, which contains eight articles (contributions 1–8) authored by experts from academia and industry. 

Nanosuspensions have been commonly used for enhancing the bioavailability of poorly soluble drugs [5,6] and long-acting injectables [7]. Wet stirred (bead) media milling (WSMM) is one of the most used processes for manufacturing drug nanosuspensions [8]. Contributions 1–3 modeled different aspects of this important process. Bitterlich et al. (contribution 1) used a stress–energy model and a steady-state energy balance model to develop a design space and control strategy of the WSMM process at the commercial manufacturing scale. Heidari et al. (contribution 2) developed a cell-based PBM to elucidate the impacts of batch size, suspension volumetric flow rate, and imperfect mixing on the evolution of particle size distribution (PSD) in a wet stirred mill. To predict particle size evolution during WSMM over a wide range of process conditions and milling scales, Clancy et al. (contribution 3) developed a practical semi-mechanistic modeling framework based on the microhydrodynamic theory of WSMM. 

The integration of advanced computational techniques such as DFT, CFD, and DEM has significantly improved our understanding of complex pharmaceutical processes. Studies like those by Middleton et al. (contribution 5) on triboelectric charging properties provide critical insights into charge transfer behaviors in pharmaceutical powders, aiding in the mitigation of electrostatic issues during manufacturing. Jin et al. (contribution 7) utilized CFD and the discrete phase model (DPM) to simulate particle deposition in nasal cavities, highlighting the impact of anatomical differences between adults and children on drug delivery, thus improving nose-to-brain drug delivery systems. Nguyen et al. (contribution 8) employed DEM to explore particle behavior upon impact, identifying various regimes that influence particle breakage and adhesion, which is crucial for optimizing pharmaceutical formulations. These studies collectively bridge significant gaps in pharmaceutical research by providing detailed mechanistic insights that enhance the efficiency and effectiveness of drug delivery and manufacturing processes.

Beyond the above-mentioned articles, other articles studied the laser ablation process used for microneedle production (contribution 4) and the twin-screw wet granulation process (contribution 6). Aldawood et al. (contribution 4) developed a multilinear regression model of microneedle diameter and height as a function of the following parameters of the laser ablation process: laser waveform, laser power, pulse width, pulse repetition, and interval between pulses. Kotamarthy et al. (contribution 6) developed a mechanistic process map to depict the effects of mixing on the time-and-space evolution of the materials inside the twin-screw granulator from liquid addition to granule formation. 

In the following section, we summarize the principal findings from each contribution in this Special Issue.

Contribution 1 implemented a control strategy and established an associated design space for WSMM of a nanocrystal suspension at a commercial manufacturing scale. In building the control strategy and design space, they used two process models: a stress–energy model for the process–product-attribute relationship of the size-reduction process and an energy balance for the temperature control of the suspension in the grinding chamber. They presented the process–product-attribute relationship with a simple, robust, and sound scientific process of controlling the mean particle size, a critical quality attribute (CQA). It is shown that this control strategy allows for flexible batch sizes, enabling different manufacturing strategies in the downstream processing of nanocrystal suspensions and adaptation to changing demands. Overall, this study exemplifies how the control strategy and design space can make use of mechanistic modeling approaches, as opposed to the sole use of design-of-experiments.

Contribution 2 developed a cell-based PBM to examine the impacts of batch size, suspension flow rate, and imperfect mixing during the production of fenofibrate nanosuspensions in a recirculating WSMM. Their study points to the criticality of incorporating a transition particle size commensurate with the notion of a grinding limit. Their experiments and the PBM predictions demonstrated that an increase in batch size entails a proportionate increase in milling time to keep the PSD invariant, and this can be achieved by keeping the same residence time in the mill for the circuit operation. Their study also showed that the suspension flow rate had an insignificant impact on the PSD. Overall, contribution 2 exemplifies how a PBM can be used for predictive purposes, apart from its common use for describing the temporal evolution of PSD. 

Contribution 3 formulated a semi-mechanistic modeling framework with elements from microhydrodynamic theory to predict particle size evolution in WSMM over a wide range of process conditions and scales. A suite of models, i.e., Models A, B, and C, with different complexity and practical utility was developed. This study demonstrated that the wide applicability of Model B in multiple case studies across drug products and mill scales. Moreover, a simplified model, Model C, was developed by fitting to data for the specific drug products and mills described in the study. As a drug-product- and mill-agnostic model, Model C was shown to provide insights into the most important mechanisms that govern milling rates. Models A–C appear to be some of the most comprehensive semi-mechanistic models for predicting the evolution of particle sizes in the WSMM literature as they explicitly consider a wide range of process–equipment–formulation parameters.

Contribution 4 proposed a new approach to fabricating microneedles using an ytterbium laser (laser ablation) and performed a design of experiments by observing the dimensions, profile surface finish, and quality of the needles. A multilinear regression model of microneedle diameter and height was developed to quantify the impacts of the waveform, laser power, pulse width, pulse repetition, and interval between pulses. This research provides a procedural framework for designing and manufacturing microneedle arrays for transdermal drug delivery applications. 

Contribution 5 employed density functional theory (DFT) to investigate the triboelectric charging of paracetamol on Al, Cu, and Ni substrates. The study found significant charge transfer from the molecule to the surface, with charge density differences revealing regions of electron enrichment and depletion around functional groups. A Hirshfeld charge analysis indicated asymmetry in charge redistribution, highlighting the different charging tendencies of atoms. This research shows that environmental and material conditions greatly influence triboelectric behavior and can improve the stability and processing of pharmaceutical powders by mitigating electrostatic issues.

Contribution 6 conducted an in-depth study on the effects of mixing dynamics in twin-screw granulation (TSG) and developed a physics-based process map to improve the understanding and control of granule quality attributes. The research focused on key intermediate parameters such as fill level, granule liquid saturation, nucleation extent, and powder wettability. These factors were analyzed for their impact on PSD, content uniformity, and granule microstructure. A significant outcome of this study was the creation of a process map illustrating the temporal and spatial evolution of materials in the TSG, providing insights into nucleation and granule growth mechanisms. This map helps predict granule quality based on material properties and process conditions, aiming to enhance continuous manufacturing efficiency in the pharmaceutical industry.

Contribution 7 conducted a computational study to compare micrometer-sized particle deposition in the olfactory regions of adult and pediatric nasal cavities, focusing on optimizing nose-to-brain drug delivery. Using CFD coupled with DPM, the authors simulated particle transport and deposition in nasal models derived from computed tomography scans. The results indicated that the olfactory region’s particle deposition fraction was minimal across both adults and children for particle sizes ranging from 1 to 100 μm, with the highest deposition fraction observed being 5.7%. The study found that the nasal cavity area in adults is approximately 1.2 to 2 times larger than in children, affecting deposition patterns. This research provides valuable insights into how nasal cavity geometry influences drug delivery efficiency, which can inform the design of intranasal therapies for neurological disorders.

Contribution 8 used DEM to study the post-impact behavior of particles with solid surfaces, focusing on spherical agglomerates composed of smaller primary particles held together by surface adhesion. The study identified five distinct behavioral regimes upon impact: rebounding, vibration, fragmentation, pancaking, and shattering. Key findings include the observation that in the rebounding regime, the coefficient of restitution decreases linearly with increasing impact velocity, leading to particle compaction. In the fragmentation regime, larger fragments showed reduced rebound velocities over time, while smaller fragments sometimes increased in velocity due to collisions. The study highlights the utility of DEM in providing detailed insights into particle behavior under various impact conditions, which are crucial for applications in pharmaceutical processing and other industries involving particulate materials. 

Overall, the articles of this Special Issue highlight the ever-increasing importance of modeling in pharmaceutical process–formulation development. While generating fundamental process–formulation insights, these articles also demonstrate how processes and formulations can be enhanced through modeling. We expect that this Special Issue will be a significant addition to the growing literature on pharmaceutical process–formulation modeling as we celebrate the 15th Anniversary of the journal *Pharmaceutics*.

## References

[1] U.S. Food and Drug Administration (2016). Applying ICH Q8(R2), Q9, and Q10 Principles to Chemistry, Manufacturing, and Controls Review.

[2] Pandey P., Bharadwaj R., Chen X., Pandey P., Bharadwaj R. (2017). Modeling of Drug Product Manufacturing Processes in the Pharmaceutical Industry. Predictive Modeling of Pharmaceutical Unit Operations.

[3] Russell A., Capece M. (2022). Pharmaceutical Process Modeling. AAPS PharmSciTech.

[4] Schenkendorf R., Gerogiorgis D.I., Mansouri S.S., Gernaey K.V. (2020). Model-Based Tools for Pharmaceutical Manufacturing Processes. Processes.

[5] Kesisoglou F., Panmai S., Wu Y. (2007). Nanosizing—Oral Formulation Development and Biopharmaceutical Evaluation. Adv. Drug Deliv. Rev..

[6] Chin W.W.L., Parmentier J., Widzinski M., Tan E.H., Gokhale R. (2014). A Brief Literature and Patent Review of Nanosuspensions to a Final Drug Product. J. Pharm. Sci..

[7] Leng D., Chen H., Li G., Guo M., Zhu Z., Xu L., Wang Y. (2014). Development and comparison of intramuscularly long-acting paliperidone palmitate nanosuspensions with different particle size. Int. J. Pharm..

[8] Malamatari M., Taylor K.M., Malamataris S., Douroumis D., Kachrimanis K. (2018). Pharmaceutical Nanocrystals: Production by Wet Milling and Applications. Drug Discov. Today.

